# A Clinical Lecture on Hæmaturia

**Published:** 1888-04

**Authors:** G. Frank Lydston

**Affiliations:** Lecturer on the Surgical Diseases of the Genito-Urinary Organs, and on Venereal Diseases, in the College of Physicians and Surgeons, Chicago, Illinois


### A CLINICAL LECTURE ON HÆMATURIA.

BY G. FRANK LYDSTON, M. D.,

Lecturer on the Surgical Diseases of the Genito-Urinary Organs, and on Venereal Diseases, in the College of Physicians and Surgeons, Chicago, Illinois.

(Reported by William Whitford.)

GENTLEMEN—The subject to which I desire to call your attention this morning is one of the greatest importance in its general relations to genito-urinary surgery. Hæmaturia, or blood in the urine, while it is seldom, if ever, a disease *per se*, constitutes one of the most prominent symptoms of a number of important affections of the genito-urinary tract. It must be remembered, however, that, although blood in the urine is not an essential disease, it is always symptomatic, we are not always able to recognize the precise pathological condition upon which it depends. The amount of blood present in the urine varies from a slight smokiness to nearly pure blood. The appearance of the urine varies considerably according to the source of the blood, the amount present and the length of time which has elapsed since the discharge of the urine. The differences in the characters of the blood present in the urine have warranted a division of cases of hæmaturia into hæmaturia pure and simple and hæmoglobinuria. In the former of these conditions the blood corpuscles originally exist intact in the urine, while in the latter they are not perceptible, the discoloration of the urine being due to a liberation of the hæmaglobin or blood pigment. Obviously the conditions which give rise to hæmaturia are apt to be of a milder character than those in which the blood is expelled with the urine in a disorganized condition.

*The diagnosis* of hæmaturia is often easily determined from the gross appearances of the urine, but in more difficult cases the microscope is necessary for the detection of the blood corpuscles. In the variety known as *hæmoglobinuria*, in which the corpuscles are broken down and only blood pigment is present, the spectroscope may be necessary to determine the presence of blood. There are several tests of a chemical character which it is well to remember. One of these, and a very popular one, is known as Day's test. It consists in adding to the suspected liquid a few drops of the alcoholic solution of *guaiac*, and then a small quantity of an ethereal solution of peroxide of hydrogen. In the presence of blood a blue color will result. The ordinary spirits of turpentine added to the urine will, with the addition of a little tincture of guaiac, also form a decidedly blue color. The diagnosis of hæmaturia having been completed, our investigations have but just begun, for it remains for us to determine the origin of the hæmorrhage, and this, as you will see later on, is in many cases a difficult matter.

*The causes of hæmaturia* are numerous. It may be due to certain general or constitutional conditions affecting the composition of the blood and the integrity of the capillary blood-vessels, as is the case in scurvy and *purpura hæmorrhagica*, the result in such cases being the form of hæmaturia known as hæmoglobinuria. It is most frequently due, however, to local disease of some portion of the genito-urinary tract, or to injury of the mucous membrane produced by the presence or passage of urinary calculi. Diseases of the kidneys—such as acute congestion or inflammation, cancer of the kidney, rupture of the kidneys from traumatism, pyelitis, renal calculus, reflex renal hyperæmia, acute inflammation from poisoning by chlorate of potassium, cantharides, turpentine and carbolic acid—are among the principal causes of hæmaturia. When the hæmorrhage is due to renal disease, epithelial or blood casts may be found by the microscope, thus clearing up the diagnosis. In scurvy, purpura hæmorrhagica, malaria, and sometimes in septicæmia, the kidney is the seat of the hæmorrhage. It may be, however, that in some cases of these conditions the hæmorrhage originates in the bladder itself. There is a form of hæmaturia, which is prevalent in tropical countries, that is produced by a parasite termed the *bilharzia hæmatobia*, after its discoverer, Theodor Bilharz, in which both the kidney and prostate are affected by the parasite, and consequently contribute to the blood in the urine. Diseases of the bladder may cause hæmaturia, the conditions which give rise to it being congestion, inflammation, vesicle calculus, villous tumor, traumatism and simple or malignant ulceration; rupture of the bladder, pathological or traumatic, is also occasionally a cause. Diseases of the prostate, such as congestion, hypertrophy, inflammation and ulceration, simple or malignant, may be also causes of the disease. Passive congestion of the prostate and bladder may result from hepatic congestion and obstruction and give rise to blood in the urine. It is sometimes the result of congestion of the prostatic plexus and straining efforts at defecation incidental to an attack of hæmorrhoids. Urethral trauma, inflammation, simple or specific, and organic stricture may give rise to hæmorrhage, which may or may not give rise to hæmaturia.

The diagnosis of the source of urinary hæmorrhage is a very important matter. As a general rule, a careful exploration of the urethra, bladder and rectum, and a careful analysis of the urine with reference to the existence of renal diseases, is necessary to determine the source of the hæmorrhage, examination of the urine involving microscopical as well as chemical and microscopical tests. The origin of many cases of hæmaturia may be revealed by a thorough knowledge of the history and constitutional condition of the patient. Much may be learned from the appearances of the urine. When the hæmorrhage is renal, the urine is smoky and the blood is uniformly diffused through it. Blood and epithelial casts are usually to be found by microscopical examination. When the hæmorrhage is vesicle or prostatic, the first urine discharged is usually clear, the last few drops being bloody, and sometimes composed of nearly pure blood, and being always wholly or in part of a bright arterial hue. In prostatic hæmorrhage the first urine expelled is apt to contain a clot or clots presenting the general *contour* and size of the prostatic sinus. This I have noticed in many cases. Under such circumstances the clot of blood is dark and semi-organized, and little or no pure blood is visible until the act of urination is nearly completed. When the hæmorrhage is urethral, blood is likely to escape independently of the act of urination, is always washed away with the first gush of urine, the last portion being quite clear, unless the hæmorrhage be very profuse. In addition to these special features, symptoms referable to disease of the particular organ involved are usually present. In some cases it may be necessary to wash out the bladder with warm water through a catheter, thus removing the blood. If the next few drachms of the urine are bloody, the hæmorrhage is probably renal. Cases of idiopathic hæmaturia have been described in which the hæmorrhage was apparently the essential disease and the cause undiscoverable. Cases of this kind are particularly apt to occur in tropical climates as a result of over-heating and extreme muscular exertion. Cases of this kind are described by Van Buren and Keyes and others. I have myself seen several cases in which there apparently existed no adequate cause for the hæmorrhage, which speedily disappeared under little or no treatment without apparent injury to the patient. I have attributed such cases to vaso-motor disturbances of the renal circulation. The condition of passive congestion of the prostate, to which allusion has already been made, is a more frequent cause of hæmaturia than has been supposed, and I have collected in my own private practice a number of cases of this kind which are of considerable interest. Three of these I will relate to you:

*Case 1.*—A young man, twenty-five years of age, was referred to me by his family physician for the relief of urethral hæmorrhage, which had occurred almost daily for six months. The doctor had suspected stricture, but as he had succeeded in passing a No. 15 English sound, he concluded that there was some disease of a more serious character existing, and therefore referred the patient to me. A history of several attacks of gonorrhœa was elicited, but further than this nothing had ever occurred which might account for the hæmorrhage. The blood escaped in the morning with great regularity, and was occasionally observed at other times during the day, especially if violent exercise had been indulged in. A fusiform clot—a sample of which was exhibited to me—generally escaped with the first gush of urine, the remainder of the flow being only slightly tinged with blood. Constipation had been a prominent symptom, and several internal piles had formed before the hæmaturia appeared. Micturition was rather frequent, but not excessively so, as the patient was not compelled to rise at night. Nocturnal emissions frequently occurred, and for several months there had been a slight gleety discharge from the urethra. The general health was only fair, digestion being disturbed the greater part of the time. On examination with the urethrameter, a stricture of large calibre was found in the penile urethra about one and a half inches from the meatus, and a slight organic contraction could be detected at the bulbo-membranous junction. Exploration of these strictures did not produce hæmorrhage. The prostate was slightly enlarged, but not tender to pressure or the passage sounds. Firm pressure upon the perineum, however, produced a sense of tension and fullness. As a preliminary to medicinal treatment, meatotomy was performed and the penile stricture cut to the calibre of 30 French, the deep stricture being treated by gradual dilatation. Internal medication consisted chiefly in remedies to relieve hepatic congestion in combination with ergot and bromide of potassium. Counter-irritation to the perineum and cold sitz baths composed the remainder of the treatment. Improvement was rapid, the hæmorrhage ceasing at the end of the first week and the other symptoms disappearing very soon thereafter.

*Case 2.*—A gentleman, thirty years of age, a patient of Dr. Frank Welles, of Chelsea, Mass. Dr. Welles had treated this patient for slight stricture and had dilated the urethra up to about 20 French, when he stopped treatment and came west. Sometime thereafter, while suffering from an attack of constipation, attended by hæmorrhoids, he noticed a small amount of blood in the urine. This recurred daily, and gradually became a source of great annoyance, although there was at no time any great loss of blood or any pain, either during micturition or at other times.

Upon examination a stricture of moderately large calibre was found at three inches from the meatus. The prostate was moderately increased in size and there were slight internal hæmorrhoids.

The treatment of this patient was precisely like that of the preceding case, with the exception that the fluid extract of hammamelis, in small doses, was substituted for the ergot. A cure was effected in six weeks.

*Case* 3.—This patient, a healthy-looking man, forty-three years of age, had never been ill until about three months prior to his consulting me, and had never had any venereal disease. At the time mentioned he had strained himself slightly in a bowling contest, and shortly thereafter began to be troubled with constipation, slight hæmorrhoids and hæmaturia. The blood in the urine had increased in quantity until it was very largely mixed with blood, fusiform clots being occasionally observed during urination. Aside from the worry consequent upon the hæmaturia, the patient was still perfectly well, appetite, sleep and strength being unimpaired, and there being absolutely no pain. For some unaccountable reason, several physicians had pronounced the case one of Bright's disease and had treated it accordingly. The symptoms appeared to warrant a diagnosis of hæmorrhage from the prostate and vesicle neck, this being determined by the absence of pain and general symptoms, the shape of the clots, the absence of vesicle irritability and urethral disease, the absence of tenderness on pressure upon the hypogastrium and perineum, the occurrence of hæmorrhage in the morning chiefly after stool and the absence of blood casts and other evidences of renal disease.

Ergot, turpentine and cholagogues, with the citrate of potassium in Vichy water, and a bread and milk diet, constituted the treatment of this case, and was perfectly successful, a cure being effected in about three weeks.

When we consider the intimate association of the hæmorrhoidal and prostatic plexuses in their relation to the neck of the bladder, prostate and anus, and the close relation of this venous net-work with the portal circulation *via* the mesenteric veins, the occurrence of such cases is hardly surprising. Considering the frequency with which a greater or less amount of urethral obstruction is superadded to simple constipation, thus necessitating more or less straining during micturition, it would indeed be remarkable if such cases were not occasionally seen.

*Treatment.*—The treatment of hæmaturia necessarily involves the proper management of its causal conditions when they are discoverable. Morbid constitutional conditions require correction, but it would be hardly proper for me, in this connection, to dwell upon the treatment of purpura hæmorrhagica and scurvy, as they are properly allotted to the instructor in another department of your college curriculum. It is also unnecessary for me to give you the minutiae of the treatment of the various local causes of the disease, inasmuch as it would be but a repetition of what will be given you in the special discussion of these diseases. In a general way I will state that inflammation of the kidneys, prostate or bladder requires hot baths, the application of leeches and hot fomentations, the administration of saline cathartics and alkaline diuretics, followed by gallic acid, matico or turpentine, in combination with any of the various demulcents which will be mentioned later on. If passive congestion or hyperæmia of any portion of the genito-urinary tract exists, the portal system requires relief by mercurial cathartics, and the local blood supply must be regulated by the administration of ergot and hammamelis. The diet should be restricted as nearly as possible to the regimen alluded to in the lecture upon genito-urinary hygiene. As a rule, catheterism should be avoided, especially if the urethra prostate or vesicle neck be the source of the hæmorrhage. Sometimes, however, the bladder becomes so completely filled with coagula as to present a strong resemblance to the gravid uterus. In such cases the fluid portion of the blood and urine should be drawn off and the coagula washed away with a double current catheter, a mild alkaline solution being best for this purpose. A digestive solution of pepsin and hydrochloric acid has been recommended for the purpose of dissolving vesicle clots, and has been claimed to be very efficacious. It may be necessary, after the removal of the clots, to attempt local hæmostasis by means of hot water or astringent irrigations. In no case should undue haste be exhibited in the attempt to evacuate clots, as it is desirable, where possible, to wait until the hæmorrhage has subsided. If decomposition of the clot occurs antiseptic irrigation is necessary. In such cases also the administration of boracic acid, salicylic acid, creasote or the iodide of potassium may assist in promoting an aseptic condition of the contents of the bladder.

The indications for the treatment of cases of hæmaturia dependent upon passive congestion of the prostate and vesicle neck are quite simple, as may be seen by the cases of that condition which have been described, and consist chiefly in the relief of venous obstruction by laxatives, especially such as act upon the liver and the removal of urethral contractures by operation or dilatation. Remedies, such as ergot and hammamelis, which act upon non-striated muscular fibre, are quite essential, but are of secondary importance. The sexual function always requires a certain amount of consideration, and the diet should be restricted to as unstimulating a regimen as possible. Neutralization of the urine should of course be accomplished as a matter of routine.

---

The *Revue de Thérapeutique* of February 1st contains a note on pulmonary tuberculosis by Professor Jaccoud. He regards it as an acute infectious disease. The only effectual method of treatment is to substitute the form which develops slowly, and may, therefore, be arrested, for the form which develops rapidly and quickly proves fatal. It is generally admitted that salicylic acid is the best agent for reducing the fever, which is the chief factor in producing the pulmonary lesions. This substance should be given in doses of two grammes daily for three days, and in doses of one and one-half grammes the three following days. The doses are then suspended for two days, after which they are resumed as before. If the fever diminishes, one gramme of salicylic acid is then administered daily.—*British Medical Journal.*

